# Molecular characteristics of advanced colorectal cancer and multi-hit *PIK3CA* mutations

**DOI:** 10.1093/oncolo/oyae259

**Published:** 2024-10-14

**Authors:** Faiza Yasin, Ethan Sokol, Neil Vasan, Dean C Pavlick, Richard S P Huang, Maureen Pelletier, Mia Alyce Levy, Lajos Pusztai, Jill Lacy, Janie Yue Zhang, Jeffrey S Ross, Michael Cecchini

**Affiliations:** Department of Medicine (Medical Oncology), Yale University, New Haven, CT 06510, United States; Yale Cancer Center, New Haven, CT 06510, United States; Foundation Medicine, Boston, MA 02210, United States; Department of Medicine (Hematology/Oncology), Columbia University, New York, NY 10032, United States; Foundation Medicine, Boston, MA 02210, United States; Foundation Medicine, Boston, MA 02210, United States; Foundation Medicine, Boston, MA 02210, United States; Foundation Medicine, Boston, MA 02210, United States; Department of Medicine (Medical Oncology), Yale University, New Haven, CT 06510, United States; Yale Cancer Center, New Haven, CT 06510, United States; Department of Medicine (Medical Oncology), Yale University, New Haven, CT 06510, United States; Yale Cancer Center, New Haven, CT 06510, United States; Department of Medicine (Medical Oncology), Yale University, New Haven, CT 06510, United States; Department of Medicine (Medical Oncology), University of Pittsburgh Medical Center, Pittsburgh, PA 15219, United States; Foundation Medicine, Boston, MA 02210, United States; Upstate Medical University, Syracuse, NY 13210, United States; Department of Medicine (Medical Oncology), Yale University, New Haven, CT 06510, United States; Yale Cancer Center, New Haven, CT 06510, United States

**Keywords:** colorectal cancer, mutations, *PIK3CA* oncogene, PI3K inhibitor

## Abstract

**Introduction:**

Approximately 20% of patients living with colorectal cancer (CRC) have activating mutations in their tumors in the *PIK3CA* oncogene. Two or more activating mutations (multi-hit) for the *PIK3CA* allele increase PI3K⍺ signaling compared to single-point mutations, resulting in exceptional response to PI3K⍺ inhibition. We aimed to identify the prevalence of *PIK3CA* multi-hit mutations in metastatic CRC to identify patients who may benefit from PI3K inhibitors.

**Methods:**

The Foundation Medicine database (Boston, MA, USA) was analyzed for patients with CRC who underwent genomic profiling on tumor DNA isolated during routine clinical care from 2013 to 2021. Molecular and clinical variables were abstracted for patients with *PIK3CA* mutations.

**Results:**

We identified 49 051 patients with CRC who underwent Foundation Medicine testing. 710/41154 (1.7%) patients had multi-hit *PIK3CA* mutations, of which 53% were male (*n* = 448) with a median age of 60. Microsatellite status was available for 697 patients with multi-hit *PIK3CA* and 17.6% (123/697) were microsatellite instability-high. Clinically relevant mutations in *KRAS* and *BRAF*^*V600E*^ were seen in 459/710 (64.7%) and 65/710 (9.1%), respectively. The 4 most common *PIK3CA* variants were H1047R (9.8%), E545K (9.2%), E542K (9.0%), and R88Q (7.1%). The most common variant pair was E542K-E545K (4.7%).

**Conclusions:**

Multi-hit mutations in *PIK3CA* are seen in 1.7% of advanced CRC, a meaningful prevalence given the high burden of CRC worldwide, and may represent a subset of patients that have enhanced sensitivity to PI3K inhibition. Future investigation regarding the clinical utility of PI3K inhibitors is warranted in multi-hit *PIK3CA* CRC.

## Teaser

This study aimed to identify the prevalence of PIK3CA multi-hit mutations in metastatic colorectal cancer to identify patients who may benefit from PI3K inhibitors.

**Implications for practice**

There have been many recent approvals for targeted therapies in advanced colorectal cancer (CRC), with ongoing advances in developing agents for molecular targets. *PIK3CA* gene mutations are well described in solid tumors with a clinically significant prevalence in CRC. There is good evidence for PI3K inhibition in breast cancer with demonstrated improved efficacy among patients with 2 or more activating mutations in the *PIK3CA* allele (“multi-hit”). We show that there is a distinct subgroup of patients with CRC that have multi-hit *PIK3CA* mutations with a co-occurring mutation profile that may be targeted for rational combination therapies.

## Introduction

Colorectal cancer (CRC) is one of the most prevalent malignancies in the United States, representing 8% of all new cancer cases with an incidence rate of 36.6 per 100 000 per year between 2016 and 2020.^1,2^ Average lifetime risk is 4.1% for men and women with variability by age, race/ethnicity, and family history.^1,3^ It remains the second most common cause of cancer-related mortality.^1,2^ While cytotoxic chemotherapy and biologics remain the backbone of preferred treatment in CRC, advances in characterizing oncogenic pathways involved in cancer pathogenesis and predictive biomarkers have led to the development and introduction of targeted therapies.^4,5^ For example, mutations in *BRAF*^*V600E*^ are seen in 10% of patients with mCRC, and the BRAF tyrosine kinase inhibitor, encorafenib, in combination with cetuximab is approved for these patients.^6^ Entrectinib and larotrectinib are TRK inhibitors approved for neurotrophic tropomyosin receptor kinase mutated mCRC.^7,8^ The anti-HER2 tyrosine kinase inhibitor (TKI) tucatinib plus HER-2 targeted trastuzumab is used for HER2-positive RAS-WT mCRC, and the anti-HER2 antibody-drug conjugate, fam-trastuzumab deruxtecan, was recently approved for all unresectable or metastatic solid tumors that are HER2 3+ without acceptable alternative treatment options.^9-12^ Furthermore, sotorasib in combination with panitumumab was recently approved for *KRAS*^*G12C*^ mutated tumors.^13^ Many studies are ongoing evaluating other potential molecular and pathway targets.

The phosphatidylinositol 3-kinase (PI3K) pathway is one that is central to cell survival, proliferation, and growth regulation and is commonly dysregulated solid tumor malignancies.^14,15^ An activating mutation in the *PIK3CA* oncogene is detected in approximately 20% of patients living with colorectal cancer (CRC).^16^*PIK3CA* codes for the catalytic subunit, p110⍺, of the phosphoinositide 3-kinase (PI3K) complex, which ultimately activates the serine/threonine protein kinase B (AKT) and mammalian target of rapamycin (mTOR) signaling pathway. *PIK3CA* activating mutations at various sites with subsequent aberrant activation of the PI3K/AKT/mTOR pathway have been described in many solid tumor malignancies, with the 3 most common hot-spot mutations being H1047R (exon 20) and E542K/E545K (exon 9).^17,18^ Much of the emerging evidence of the PI3K pathway as an actionable target for treatment has been in hormone-receptor positive (HR+), HER2-negative breast cancer.^19-22^ Early-phase clinical trials have shown that PI3K inhibition alone has low activity, but rational combinations such as alpelisib with fulvestrant result in clinically meaningful benefits.^20,23^ Moreover, when selecting patients with 2 activating mutations (“multi-hit”) in the *PIK3CA* allele, improved survival is described in multiple PI3K inhibitor trials for breast cancer.^21,24,25^ The totality of the pre-clinical and clinical data suggests that multi-hit *PIK3CA* mutated tumors are more sensitive to PI3K inhibitors, and identifying tumors beyond breast cancer with multi-hit *PIK3CA* mutations may broaden the patient population that may benefit from this therapeutic approach.

Due to the pre-clinical and clinical data supporting multi-hit *PIK3CA* as an actionable target and the high prevalence of *PIK3CA* mutations in CRC, we aim to identify the prevalence of the mutation, co-occurring mutations, and patient characteristics of multi-hit *PIK3CA* in CRC. We performed a retrospective cohort study using the Foundation Medicine database to identify the molecular and clinical characteristics of patients with multi-hit *PIK3CA* CRC.

## Methods

### Design

Approval for this study was obtained from the Western Institutional Review Board (Protocol No. 20152817). The Foundation Medicine database was queried from 2013 to 2021 to identify patients with advanced CRC tumors with at least 2 activating mutations in *PIK3CA* and those which were *PIK3CA* wild-type. Pathology reports and clinical variables reported to Foundation Medicine by the ordering clinician were extracted for analysis.

### Molecular analysis and clinical data

Tissue-based comprehensive genomic profiling (CGP) of either a primary colorectal tumor or a metastatic site biopsy was performed in a Clinical Laboratory Improvement Amendments-certified, CAP (College of American Pathologists)-accredited laboratory (Foundation Medicine Inc., Cambridge, MA, USA) on all-comers with advanced colorectal cancer during the course of routine clinical care from 2013 to 2021. Hybrid capture was carried out for at least 324 cancer-related genes, including *PIK3CA* (**Figure S1**).

At least ≥50 ng DNA was extracted from 40 µm of formalin-fixed, paraffin-embedded (FFPE) sections. FoundationOne CDx hybrid capture-based sequencing using adaptor ligation-based libraries was utilized, with mean coverage depth >600×. Base substitutions, insertions, deletions (short variants; SV), rearrangements, and copy number changes were assessed.^26-28^ To help visualize the sequencing data results, an oncoprint plot was generated with the online tools of the cbio portal as described by Gao et al and Cerami et al.^29,30^ Tumor mutational burden was determined on 0.8-1.2 Mb of sequenced DNA using a mutation burden estimation algorithm that, based on the genomic alterations detected.^31^ Assessment of microsatellite instability was performed from DNA sequencing, as previously described.^32^ The next-generation sequencing-based “microsatellite instability score” was translated into microsatellite instability high, microsatellite instability intermediate, or microsatellite stable by unsupervised clustering of specimens for which microsatellite instability status was previously assessed via gold standard methods.^32^ PD-L1 expression was determined on subsets of the tumors using the DAKO 22C3 assay with low positive tumor cell scoring defined as 1%-49% staining and high positive tumor cell scoring defined as ≥50% staining.

Clinical variables evaluated included age, sex, site, and metastatic biopsy site. The Foundation Medicine database includes clinically advanced tumors that are primarily stage IV, with fewer than 1% of cases that are not metastatic at the time of tumor profiling.

### Statistical analysis

Molecular results and clinical characteristics are reported using descriptive statistics. The software package R, version 4.3.3 (R Foundation for Statistical Computing), was used for statistical analyses. The Fisher’s exact test was used to calculate *P*-values for the individual mutations which were evaluated as categorical variables.

## Results

**Figure 1.**
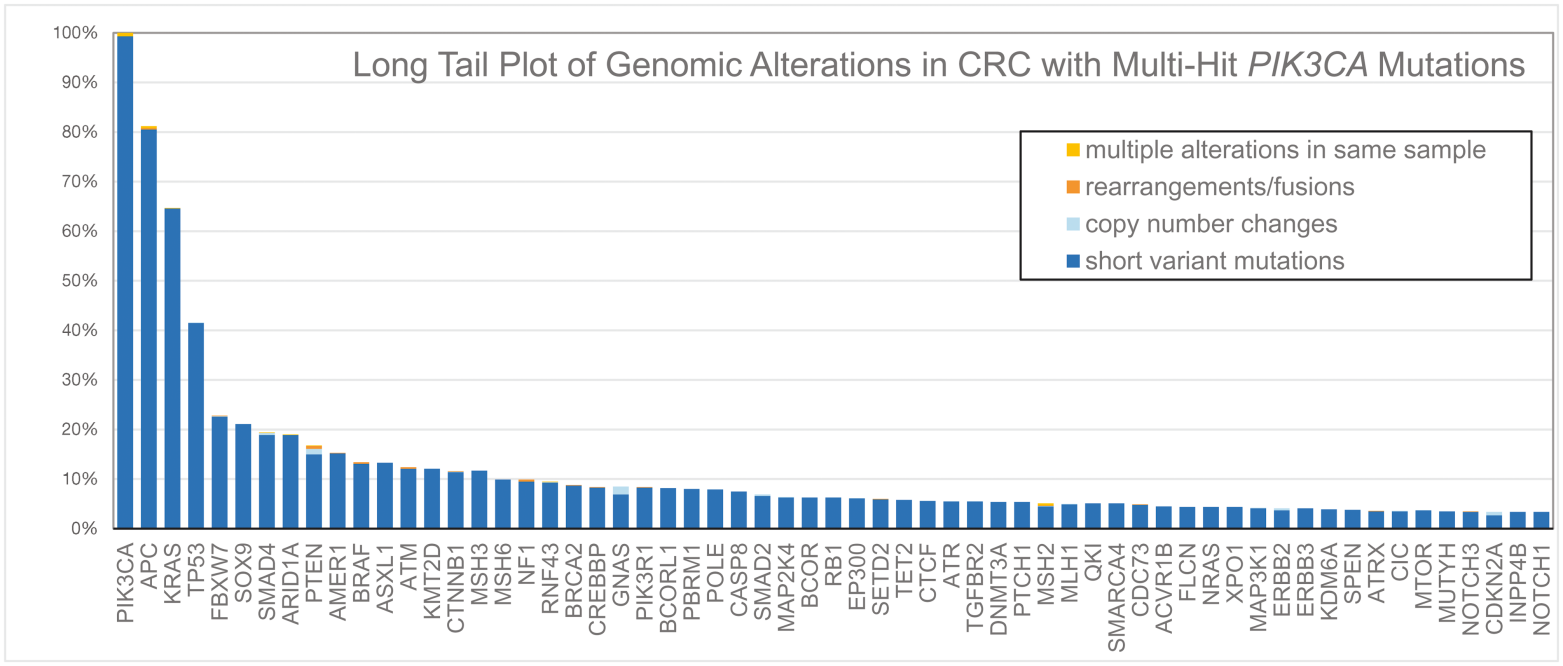
Long tail plot of genomic alterations in CRC with multi-hit PIK3CA mutations.

**Figure 2.**
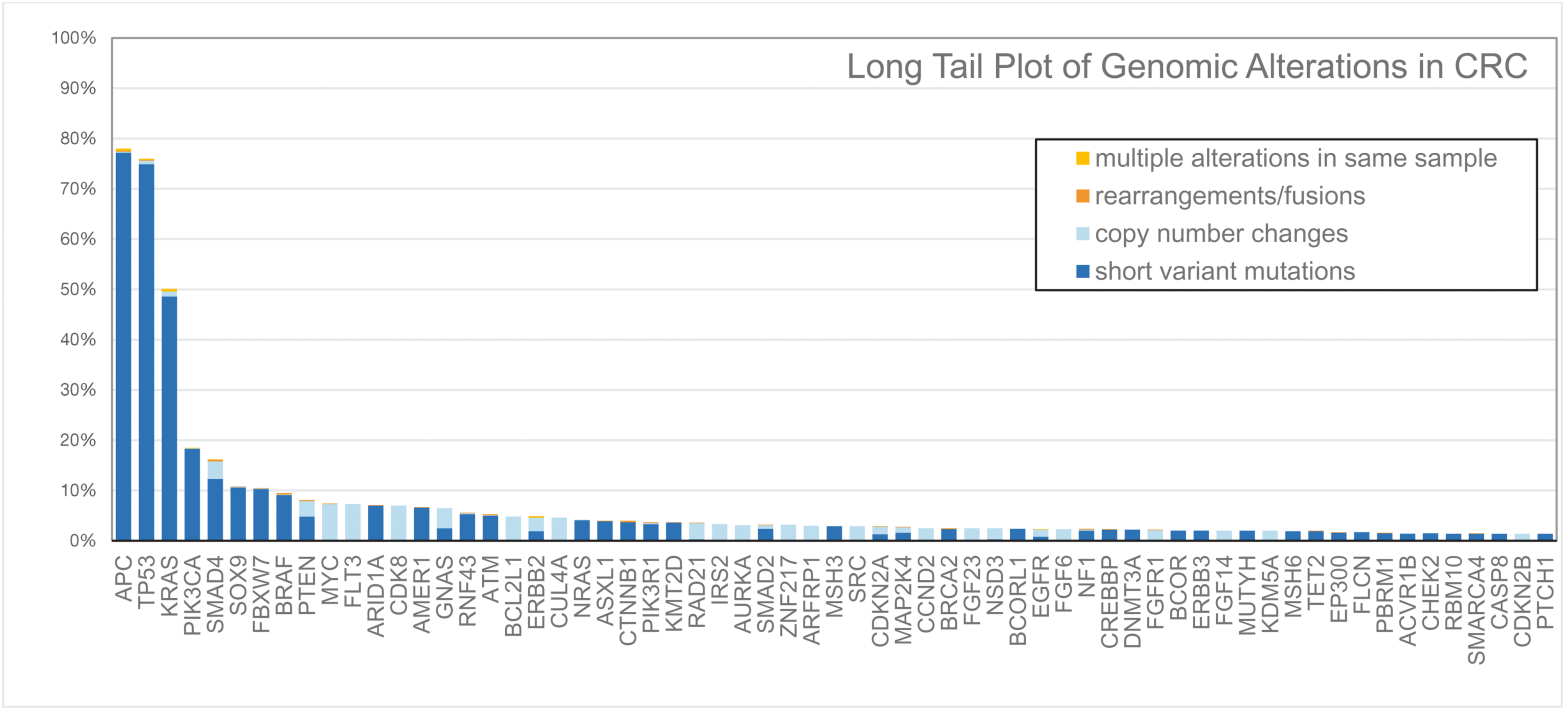
Long tail plot of genomic alterations in CRC.

We identified 49 051 patients with advanced CRC that underwent tissue-based comprehensive genomic profiling by Foundation Medicine from January 2013 through December 2021. There were 846 total cases identified with multi-hit *PIK3CA* mutations, and 39 998 cases without any *PIK3CA* mutations. Additional clinical and molecular data was available for 41 154 patients, of which 710 (1.72%) had multi-hit *PIK3CA* mutations (**Figure 1**), and 7627 (19%) had any deleterious *PIK3CA* mutation (**Figure 2**).

The primary colon tumor was used for sequencing in 70% (590/846) of cases with multi-hit *PIK3CA* mutations and a metastatic site was used in 30% (256/846) of the cases. Clinical characteristics of the group with multi-hit *PIK3CA* mutations and the *PIK3CA* wild-type group are described in **Table 1**. Patients with *PIK3CA* multi-hit mutations were 53% (448/846) male with a median age of 60 years (interquartile range 50-70). The microsatellite status was available for 697 patients with multi-hit *PIK3CA* CRC, of which 17.65% (123/697) were microsatellite instability-high (MSI-high) compared to 3.46% (1119/32,335) of wild-type *PIK3CA* CRC (*P* < .001). PDL-1 status was available for 194 patients with multi-hit *PIK3CA* CRC, of which 16.5% (32/194) of patients were PDL-1 positive compared to 11.43% (944/8,265) of the wild-type *PIK3CA* CRC (*P* = .040). These data are described in **Table 2**.

**Table 1.** Baseline characteristics.

	Multi-hit PIK3CA mutations *n* (%)	No PIK3CA mutations *N* (%)
Total number of cases, *n*	846	39 998
Number of cases analyzed, *n*	710	32 964
Sex		
Female	398 (47%)	17 599 (44%)
Male	448 (53%)	22 399 (56%)
Median age (range)	60 (18-89+)	59 (8-89+)
Primary site colon tissue used for genomic profiling	590 (70%)	21 199 (53%)
CRC metastasis biopsy used for genomic profiling	256 (30%)	18 799 (47%)
Driver alterations/ sample	13.4	5.9

**Table 2.** Immuno-oncology drug biomarkers.

	Multi-hit PIK3CA mutations *n* (%)	No PIK3CA mutations *N* (%)	*P*-value
MSI high frequency			
Cases evaluated, *n*	697	32 335	
MSI-high	123 (17.6%)	1119 (3.5%)	<.001
*CD274* (PDL-1) amplification	4 (0.6%)	99 (0.3%)	.173
*STK11* inactivating genomic alteration	7 (1.0%)	231 (0.7%)	.358
*MDM2* amplification	1 (0.1%)	99 (0.3%)	.727
*PBRM1*	57 (8.0%)	396 (1.2%)	<.001
Median TMB	5.2	3.5	
TMB ≥ 10	225 (31.7%)	2185 (6.6%)	<.001
TMB ≥ 20	188 (26.5%)	1190 (3.6%)	<.001
PD-L1 status			
Cases evaluated, *n*	194	8265	
PD-L1 positive	32 (16.5%)	945 (11.4%)	.040
PD-L1 low positive	30 (15.5%)	848 (10.3%)	
PD-L1 high positive	2 (1.0%)	386 (1.2%)	

**Figure 3.**
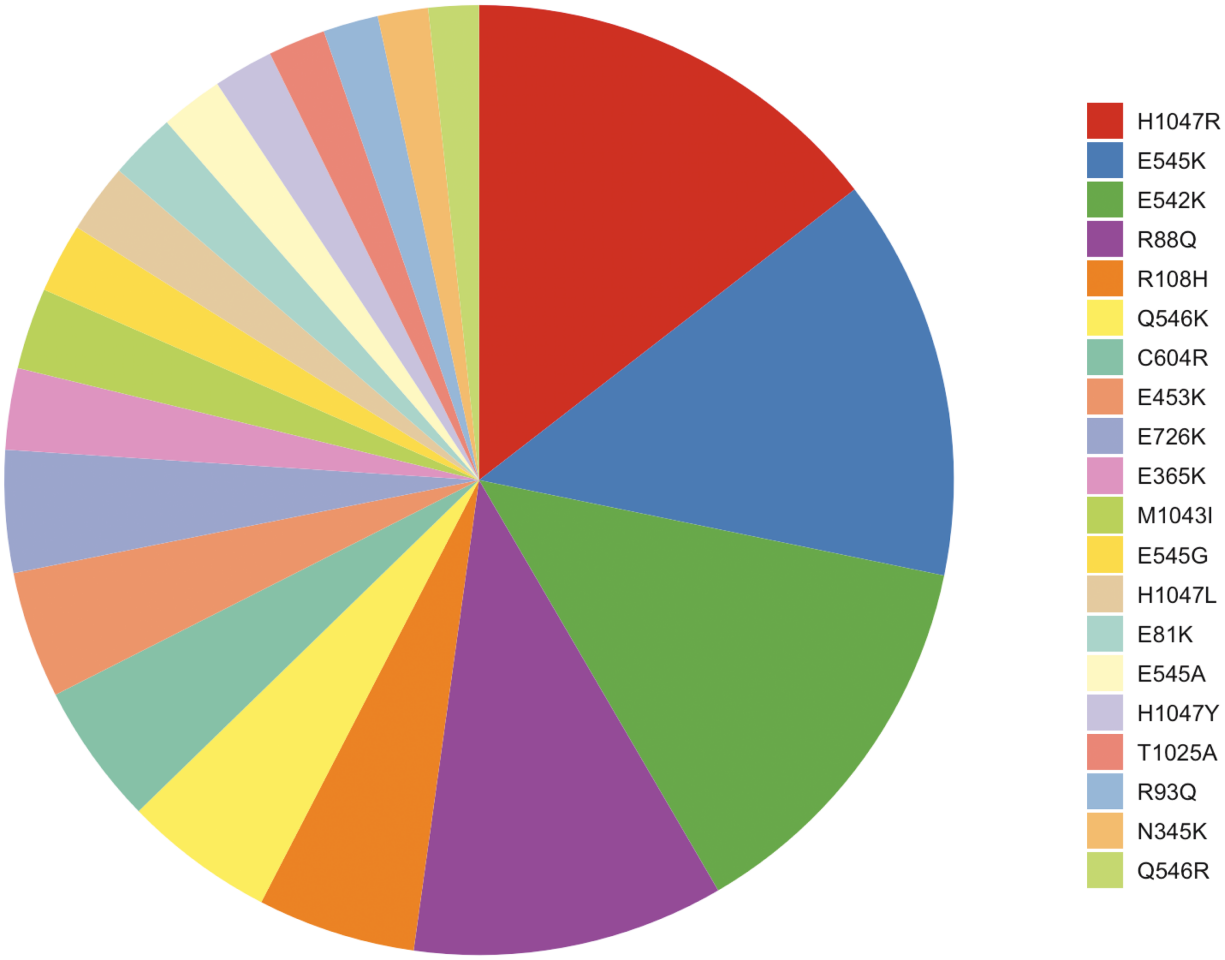
PIK3CA variant allele frequencies in multi-hit PIK3CA cases, top 20.

The multi-hit tumors were enriched for higher TMB, with TMB > 20% in 26.5% (188/710) of multi-hit *PIK3CA* tumors compared to 3.61% (1190/32,964) in patients without any *PIK3CA* mutations (*P* < .001) (**Table 3**)**.** Multi-hit *PIK3CA CRC* tumors showed a higher prevalence of mutations in DNA damage response compared to unmutated *PIK3CA* tumors notably in *BRCA2* (8.90% vs 2.00%, *P* < .001), and *ATM* (12.40% vs 4.60%, *P* < .001). The multi-hit group was also enriched for mutations in MAPK pathway with high prevalence of co-occurring *KRAS* (64.70% vs 46.90%, *P* < .001) and *BRAF*^*V600E*^ (9.10% vs 6.67%, *P* = .012) mutations. A higher prevalence of concurrent chromosomal remodeling mutations and mismatch repair proteins was also seen in the multi-hit *PIK3CA* group. Mutations in *TP53* were less common in the multi-hit *PIK3CA* mutated group compared to the wild-type *PIK3CA* group (41.60% vs 80.60%, *P* < .001). The 4 most common *PIK3CA* variants detected in our sample were H1047R (9.8%), E545K (9.2%), E542K (9.0%) and R88Q (7.1%) (**Table 4**, **Figure 3**). The most common variant pair was E542K with E545K in 4.7% of multi-hit cases.

**Table 3.** Genomic profiling data by category.

	Multi-hit PIK3CA mutations *n* (%)	No PIK3CA mutations *N* (%)	*P*-value
**CRC-related tumor suppressor genes**			
*APC*	577 (81.2%)	25 481 (77.3%)	.013
*SMAD4*	138 (19.5%)	5307 (16.1%)	.020
*RNF43*	68 (9.6%)	1714 (5.2%)	<.001
*CREBBP*	60 (8.5%)	593 (1.8%)	<.001
**Cell adhesion and Wnt signaling**			
*CDH1*	18 (2.5%)	363 (1.1%)	<.001
*AMER1*	109 (15.4%)	1846 (5.6%)	<.001
*CTNNB1*	83 (11.7%)	1088 (3.3%)	<.001
**C** **ell cycle regulatory genomic alterations**			
*TP53*	295 (41.6%)	26 569 (80.6%)	<.001
*CDKN2A*	24 (3.4%)	923 (2.8%)	.357
*CDKN2B*	6 (0.8%)	494 (1.5%)	.206
*CDK4*	1 (0.1%)	66 (0.2%)	1
*CDK6*	3 (0.4%)	264 (0.8%)	.388
*CDK8*	0 (0.0%)	2571 (7.8%)	<.001
*CCND1*	7 (1.0%)	396 (1.2%)	.728
*RB1*	45 (6.3%)	330 (1.0%)	<.001
**Lynch syndrome associated genes**			
*MSH3*	83 (11.7%)	725 (2.2%)	<.001
*MSH2*	37 (5.2%)	198 (0.6%)	<.001
*MSH6*	70 (9.9%)	429 (1.3%)	<.001
*MLH1*	36 (5.1%)	264 (0.8%)	<.001
*PMS2*	0 (0.0%)	264 (0.8%)	.008
**Histones, chromosomal, and chromatin-related genomic alterations**			
*TERT*	20 (2.8%)	330 (1.0%)	<.001
*ASXL1*	94 (13.3%)	1055 (3.2%)	<.001
*ARID1A*	135 (19.0%)	2011 (6.1%)	<.001
*SMARCA4*	36 (5.1%)	429 (1.3%)	<.001
*KMT2D*	86 (12.1%)	956 (2.9%)	<.001
**RAS-RAF-MAPK pathway genomic alterations**			
*KRAS* (all)	459 (64.7%)	15 460 (46.9%)	<.001
*KRAS G12C*	22 (3.0%)	1098 (3.3%)	.832
*NRAS*	31 (4.4%)	1450 (4.4%)	1
*MAP3K1*	30 (4.2%)	264 (0.8%)	<.001
*BRAF* mutations (all)	95 (13.4%)	3066 (9.3%)	<.001
*BRAF* V600E	65 (9.1%)	2199 (6.7%)	.012
**PI Kinase and MTOR pathway genomic alterations**			
*PIK3CA*	710 (100.0%)	0 (0.0%)	0
*PTEN*	119 (16.8%)	2472 (7.5%)	<.001
*FBXW7*	162 (22.8%)	3099 (9.4%)	<.001
*NF1*	70 (9.9%)	692 (2.1%)	<.001
*TSC1*	10 (1.4%)	165 (0.5%)	.004
*TSC2*	14 (2.0%)	330 (1.0%)	.021
*AKT1*	10 (1.4%)	363 (1.1%)	.464
**DNA damage response associated genomic alterations**			
*POLE*	56 (7.9%)	66 (0.2%)	<.001
*BRCA1*	18 (2.5%)	363 (1.1%)	.002
*BRCA2*	63 (8.9%)	659 (2.0%)	<.001
*ATM*	88 (12.4%)	1516 (4.6%)	<.001
**Receptor tyrosine kinase targetable genomic alterations**			
*ERBB2* amplification	3 (0.4%)	956 (2.9%)	<.001
*ERBB2* sequence mutation	26 (3.7%)	560 (1.7%)	<.001
*ERBB3* sequence mutation	29 (4.1%)	560 (1.7%)	<.001
*EGFR*	16 (2.3%)	857 (2.6%)	.719
*FGFR1*	7 (1.0%)	791 (2.4%)	.012
*FGFR2*	13 (1.8%)	165 (0.5%)	<.001
*FGFR3*	2 (0.3%)	66 (0.2%)	.656
*FGFR4*	4 (0.6%)	66 (0.2%)	.060
*ALK* fusion	0 (0.0%)	33 (0.1%)	1
*ROS1* fusion	0 (0.0%)	33 (0.1%)	1
*RET* fusion	0 (0.0%)	66 (0.2%)	.651
*NTRK* fusion	0 (0.0%)	66 (0.2%)	.651
*FLT3*	9 (1.3%)	2604 (7.9%)	<.001
*MET*	11 (1.6%)	330 (1.0%)	.178
*KIT*	11 (1.6%)	132 (0.4%)	<.001
Hedge-hog pathway			
*PTCH1*	38 (5.4%)	363 (1.1%)	<.001
*SMO*	14 (2.0%)	99 (0.3%)	<.001
**Transcription factor genomic alterations**			
*SOX9*	150 (21.1%)	3132 (9.5%)	<.001
*MYC*	15 (2.1%)	2736 (8.3%)	<.001
**Emerging potentially genomic alterations**			
*NOTCH1*	24 (3.4%)	363 (1.1%)	<.001
*NOTCH2*	18 (2.5%)	264 (0.8%)	<.001
*MTAP*	9 (1.3%)	461 (1.4%)	1

**Table 4.** Multi-hit *PIK3CA* colorectal cancer; *PIK3CA* variant allele frequencies.

PIK3CA variant	*n*	%
H1047R	178	9.76
E545K	168	9.22
E542K	164	9.00
R88Q	130	7.13
R108H	66	3.62
Q546K	63	3.46
C604R	59	3.24
E453K	53	2.91
E726K	51	2.80
E365K	34	1.87
M1043I	34	1.87
E545G	29	1.59
H1047L	29	1.59
E81K	28	1.54
E545A	26	1.43
H1047Y	25	1.37
T1025A	24	1.32
R93Q	23	1.26
N345K	21	1.15
Q546R	21	1.15

Co-occurring genomic mutations for multi-hit *PIK3CA* patients, and *PIK3CA* wildtype patients are described in **Tables 2** and **3**.

## Discussion

*PIK3CA* gene mutations are well-described in multiple solid tumors, including CRC.^15^ In concordance with established literature, we find that *PIK3CA* single-gene alterations are seen in 19% of patients with advanced colorectal cancers.^18^ To date, PI3K inhibitors have not been approved for use in colorectal cancer, however, pre-clinical data and clinical data in breast cancer demonstrate that patients with multi-hit *PIK3CA* mutations as compared to single-hit *PIK3CA* have enhanced PI3K activity, and substantial clinical benefit.^18,20,21,33,34^ Therefore identifying patients in CRC who may benefit from PI3K inhibitors addresses an unmet need. A summary table of currently actionable genomic alterations in metastatic colorectal cancer is included in Table S1 to put this genomic alternation in the context of the current treatment landscape.

Our manuscript represents the largest data regarding *PIK3CA* mutation prevalence in advanced colorectal cancer, demonstrating that *PIK3CA* multi-hit mutations are present in 1.7% of advanced CRC. This is similar to previous studies that reported the prevalence of multi-hit *PIK3CA* mutations across tumor types.^18,35^ In a highly prevalent disease such as CRC a 1.7% prevalence is a clinically meaningful subset of patients who may have enhanced sensitivity to PI3K inhibitors via increased PI3K⍺ signaling, leading to improved treatment responses.^18,35^

The 3 most common *PIK3CA* variants described in the literature are E545K and E542K in the helical domain, and H1047R in the kinase domain, across tumor types, which is concordant with our sample findings.^36^

In our sample, patients with multi-hit *PIK3CA* CRC have a genomic profile of co-occurring mutations that is distinct from single-hit *PIK3CA* mutated CRC and *PIK3CA* wildtype CRC; they are enriched for DNA damage response mutations, MAPK pathway mutations, and chromosomal remodeling mutations. This provides an opportunity for combination therapies by targeting different components of the PI3K pathways in addition to other relevant pathways. There are ongoing clinical trials evaluating PI3K⍺ inhibitors in combination with other targeted therapies, including the phase 3 study evaluating the novel *PIK3CA* inhibitor, Inavolisib, in combination with Palbociclib and fulvestrant in *PIK3CA*-mutant, HR+, Her2 negative breast cancer (NCT04191499).^37-39^ A large global study is ongoing of targeted therapies based on genomic alterations across all unresectable, locally advanced, and metastatic solid tumors, including assessment of Inavolisib on all multi-hit *PIK3CA* mutated tumors, including CRC (TAPISTRY NCT04589845).^40^ There are also several early phase trials ongoing of PI3K inhibitors for treatment of CRC including MEN1611 in combination with cetuximab in *PIK3CA* mutated mCRC (NCT04495621). Results from these cohorts will further validate which subsets of patients benefit most from PI3K-targeted therapies. It is of note that a substantial portion of those with multi-hit *PIK3CA* mutations also had RAS/RAF mutations: 64.7% with any *KRAS* mutation (3% with *KRAS*^*G12C*^), 13.4% with any *BRAF* mutation (9.1% with *BRAF*^*V600E*^), and 4.4% with *NRAS* mutation. Co-occurring RAS/RAF mutations may affect inhibiting the *PIK3CA* pathway with monotherapy PI3K inhibitors, and combination strategies may be indicated for patients with combined *PIK3CA* and MAP kinase activation.

Additionally, we identified multi-hit *PIK3CA* mutated tumors to have higher TMB and are more likely to be MSI-high. Moreover, pre-clinical data demonstrates the improved anti-tumor effect of the combination PI3K inhibitor and immunotherapy.^41^ However, it remains unknown whether multi-hit *PIK3CA* tumors that are TMB high and/or are MSI-high are driven by the *PIK3CA* mutations or are primarily passenger mutations driven by high TMB. Thus it is possible that PI3K inhibition may be less effective in this subgroup, especially in MSI-high patients with high TMB > 20 who also have excellent alternative treatment options with immune checkpoint inhibitors. There are ongoing trials evaluating concurrent PI3K inhibition with immunotherapy to better understand this rational combination; including a phase I/II study of the pan-class PI3K inhibitor, copanlisib, with nivolumab and ipilimumab in patients with PI3K mutated advanced solid tumors (NCT04317105). Another multicenter phase I/II study of copanlisib with nivolumab is ongoing specifically in relapsed/refractory mismatch-repair proficient advanced colorectal cancer, with early results demonstrating durable responses and meeting the primary endpoint of partial response or complete response in the *PIK3CA*-mutated cohort (NCT03711058).^42^

Finally, the adverse effect profile and toxicities of PI3K inhibitors, most notably by inhibition of insulin signaling resulting in high-grade hyperglycemia, have previously been described to be dose and treatment-limiting.^18-20^ Complete inhibition of the pathway may be possible with improved therapeutic effect if on-target toxicities can be managed or optimized.^34^ Optimal patient selection by biomarkers may thus allow for lower therapeutic doses and improved toxicity profile, overall improving the efficacy of PI3K inhibitors.

Our study has several limitations, including the retrospective nature of analysis and data. Complete clinical data were not available for review to comprehensively understand disease burden, sidedness, medical co-morbidities, and disease risk factors. Incomplete data were also excluded which may introduce some selection bias in our sample. Our data also cannot identify with certainty that the multi-hit *PIK3CA* mutations in our sample occur on the same allele. Despite these limitations, the sample size was large, included multiple treatment settings, and allowed for the assessment of multiple co-occurring mutations using a large genomic analysis panel.

In summary, we demonstrate that there is a distinct subset of patients with metastatic mCRC who have multi-hit PI3K mutations in addition to a distinct constellation of co-occurring mutations that may be potential targets for rational combination therapies. Based on these observations, it may be reasonable for future clinical trials to select multi-hit *PIK3CA* tumors that may derive greater benefit from PIK3 inhibition and potentially require lower therapeutic doses to achieve effect. It is reasonable to posit future potential combination drug trials with PI3K inhibition in patients with multi-hit *PI3KCA* mCRC in addition to other distinct targets that were found to be enriched in this group. This may also be a group of individuals who would benefit from the addition of a PI3K inhibitor to the current standard chemotherapy plus biologic therapy or immunotherapy, which further investigations may evaluate.

## Supplementary material

Supplementary material is available at *The Oncologist* online.

oyae259_suppl_Supplementary_Figure_S1_Table_S1

## Data Availability

The datasets used and/or analyzed during the current study are available on reasonable request.
