# Supplementary material for: Molecular characteristics of advanced colorectal cancer and multi-hit PIK3CA mutations
Source: Oncologist. 2024 Oct 14;29(12):1059–67. doi: 10.1093/oncolo/oyae259 (PMC11630746; doi:10.1093/oncolo/oyae259)
Supplement: oyae259_suppl_Supplementary_Figure_S1_Table_S1 [file oyae259_suppl_supplementary_figure_s1_table_s1.docx]

**
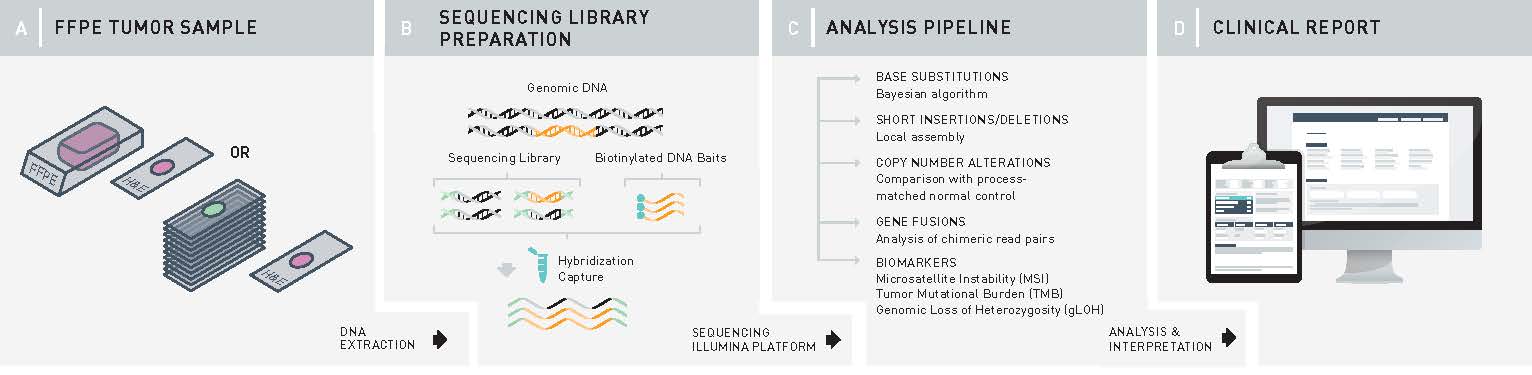
**

**Supplementary Figure 1: Comprehensive genomic sampling and hybrid capture**

**Supplemental Table 1: Targeted agents approved for metastatic colorectal cancer (mCRC) by genomic alteration**

| **Genomic alteration** | **Prevalence in mCRC** | **Currently approved therapies** |
| --- | --- | --- |
| *BRAF^V600^* mutated | 10% | Encorafenib plus cetuximab^6^ |
| *NTRK* gene fusion-positive | Less than 1%^43^ | Entrectinib^8^  Larotrectinib^7^ |
| HER2 amplified and *RAS* and *BRAF-*wild type | 2-5%, up to 10% | Trastuzumab plus Tucatinib^9^ |
| HER-2 amplified (3+ by IHC) | 2-5% | Fam-trastuzumab deruxtecan-nxki^10-12^ |
| *KRAS^G12C^* mutated | 3-4%^44^ | Sotorasib plus panitumumab^13^  Adagrasib plus cetuximab^44^ |
| *RET* gene-fusion | Less than 1%^45^ | Selpercatinib^46^ |

References

43. Karan C, Tan E, Sarfraz H, et al. Characterization of NTRK alterations in metastatic colorectal cancer. Journal of Clinical Oncology 2022;40:e15569-e.

44. Yaeger R, Weiss J, Pelster MS, et al. Adagrasib with or without Cetuximab in Colorectal Cancer with Mutated KRAS G12C. N Engl J Med 2023;388:44-54.

45. Nagasaka M, Brazel D, Baca Y, et al. Pan-tumor survey of RET fusions as detected by next-generation RNA sequencing identified RET fusion positive colorectal carcinoma as a unique molecular subset. Transl Oncol 2023;36:101744.

46. Duke ES, Bradford D, Marcovitz M, et al. FDA Approval Summary: Selpercatinib for the Treatment of Advanced RET Fusion-Positive Solid Tumors. Clin Cancer Res 2023;29:3573-8.
